# Gaussian process regression for ultrasound scanline interpolation

**DOI:** 10.1117/1.JMI.9.3.037001

**Published:** 2022-05-17

**Authors:** Alperen Degirmenci, Robert D. Howe, Douglas P. Perrin

**Affiliations:** a Harvard University, John A. Paulson School of Engineering and Applied Sciences, Cambridge, Massachusetts, United States; b Boston Children’s Hospital, Boston, Massachusetts, United States; c Harvard Medical School, Boston, Massachusetts, United States

**Keywords:** ultrasound, interpolation, Gaussian process, machine learning

## Abstract

**Purpose:**

In ultrasound imaging, interpolation is a key step in converting scanline data to brightness-mode (B-mode) images. Conventional methods, such as bilinear interpolation, do not fully capture the spatial dependence between data points, which leads to deviations from the underlying probability distribution at the interpolation points.

**Approach:**

We propose Gaussian process (GP) regression as an improved method for ultrasound scanline interpolation. Using ultrasound scanlines acquired from two different ultrasound scanners during *in vivo* trials, we compare the scanline conversion accuracy of three standard interpolation methods with that of GP regression, measuring the peak signal-to-noise ratio (PSNR) and mean absolute error (MAE) for each method.

**Results:**

The PSNR and MAE scores show that GP regression leads to more accurate scanline conversion compared to the nearest neighbor, bilinear, and cubic spline interpolation methods, for both datasets. Furthermore, limiting the interpolation window size of GP regression to 15 reduces computation time with minimal to no reduction in PSNR.

**Conclusions:**

GP regression quantitatively leads to more accurate scanline conversion and provides uncertainty estimates at each of the interpolation points. Our windowing method reduces the computational cost of using GP regression for scanline conversion.

## 1 Introduction

Ultrasound imaging is one of the safest, cheapest, and fastest medical imaging modalities. It is widely used in many clinical applications, such as image-guided interventions, as a diagnostic as well as a therapeutic tool. Ultrasound image resolution and accuracy are important factors that can impact the outcome of such procedures.

The ultrasound transducer transmits and receives radio frequency data along scanlines. This scanline data are then processed, undergoing operations such as time gain compensation, low-pass filtering, envelope detection, and log compression.^1^ Finally, the processed scanlines (A-mode) are interpolated such that a dense two-dimensional (2D) image [brightness-mode (B-mode)] can be displayed to the clinician.

In a probabilistic framework, the data along the scanlines can be considered as samples drawn from an underlying probability distribution, and then the goal of the interpolation step during scan conversion is to approximate from this underlying distribution the values of pixels for which we do not have a measurement. Commonly used interpolation methods, such as bilinear interpolation, only use local information from a few neighboring observations, which can lead to inaccurate estimates. Methods that take into account more observations can better infer the underlying distribution, leading to more accurate interpolation.^2^

In this paper, we use Gaussian process (GP) regression to improve the accuracy of ultrasound scanline conversion. In GP regression, a Gaussian distribution function is fit to each observation point. A covariance function is used to compute the spatial correlation between observations. The parameters of this function can be tuned to capture different characteristic length scales that are specific to each dimension of the data. This adaptability is useful in ultrasound imaging, since observations along a scanline are dense, whereas the number of scanlines is small in comparison.

### 1.1 Previous Work on Improving Ultrasound Image Quality

Most work on ultrasound image enhancement has focused on speckle reduction^3^^,^^4^ and denoising.^5^ An anisotropic spatiotemporal smoothing filter was developed^6^ to improve scan conversion accuracy. Spline interpolation for three-dimensional (3D) ultrasound volume compounding was explored in Ref. 7. Kriging (another name for GP regression in geostatistics) was used for 3D ultrasound reconstruction from multiple scan converted 2D slices in Ref. 8. A random field approach was used in Ref. 9 to construct confidence maps for ultrasound images, which are then used to improve 3D ultrasound interpolation accuracy. A comprehensive review of interpolation for 3D ultrasound compounding can be found in Ref. 10.

Recent work has applied deep-learning methods to ultrasound imaging,^11^^–^^13^ such as dynamic beam forming, adaptive spectral Doppler processing, improved noise suppression, and super-resolution. However, these techniques are yet far from clinical adoption, since acquiring high-quality ground truth data at massive scales required for model training and ensuring generalization to unseen anatomy remains a challenge in developing and validating such deep-learning-based solutions.

GP regression was previously used in other imaging modalities: single-image super-resolution was explored^14^ for natural images^15^; explored magnetic resonance imaging (MRI)^16^ used the variance estimates from GP regression to improve MRI image registration. To the best of our knowledge, GP regression has previously not been applied to ultrasound scanline interpolation.

## 2 Methods

Let x represent the spatial positions of our observations y along the scanlines, and f(x)=y be a nonlinear function that maps x to y. The core assumption in our work is that f can be approximated by a Gaussian process, allowing us to perform scanline interpolation by querying the process at the desired interpolation coordinates, $x_{*}$.

### 2.1 Gaussian Process Regression

A GP is a collection of random variables, any finite number of which have a joint Gaussian distribution.^17^ A GP is completely specified by its mean function, m(x), and covariance function, $k(x,x^{\prime })$, represented as $f(x)∼\mathrm{GP}(m(x),k(x,x^{\prime }))$. In practice, a zero-mean function is commonly used when we have limited knowledge of f.^18^ Observations are considered as random variables that are drawn from a multivariate normal distribution

$$[\begin{array}{c} y\\ y_{*} \end{array}]∼N(0,[\begin{array}{cc} K+\sigma_{n}^{2}I & K_{*}\\ K_{*}^{T} & K_{**} \end{array}]),$$ (1)

where y is a vector of observed scanline intensities, $y_{*}$ is a vector of intensities we seek to estimate at the interpolation locations, and $\sigma_{n}^{2}$ is the observation noise. The covariance matrix is a positive semi-definite symmetric matrix, where K=k(x,x) is the covariance between observations, $K_{*}=k(x,x_{*})$ is the covariance between observations and interpolation points, and $K_{**}=k(x_{*},x_{*})$ is the covariance between interpolation points. The details of the covariance function will be discussed in Sec. 2.3.

The intensity vector $y_{*}$ is estimated at the interpolation locations $x_{*}$ as

$${\overset{¯}{y}}_{*}=K_{*}^{T}{\left(K+\sigma_{n}^{2}I\right)}^{-1}y.$$ (2)

Concerns regarding the computational costs of evaluating Eq. (2), namely, inverting the covariance matrix, are addressed in Sec. 2.6.

The variance of the regressed values can also be computed, based on the distance between the observations and the interpolation locations, using

$$V[y_{*}]=K_{**}-K_{*}^{T}{\left(K+\sigma_{n}^{2}I\right)}^{-1}K_{*}.$$ (3)

When computing Eqs. (2) and (3), we standardize the inputs to account for the zero-mean function used in Eq. (1), as well as to avoid numerical instabilities and rounding errors in floating-point arithmetic operations.

### 2.2 Scanline Conversion—Parallel and Diverging Scanlines

In ultrasound transducers, the scanlines can be either parallel to each other (e.g., from a linear-array probe), or diverging, spreading out radially from the source (e.g., from a phased-array or curvilinear probe). With parallel scanlines, the scanline data form a grid in Cartesian coordinates, and it is straightforward to interpolate and display them.

**Fig. 1:**
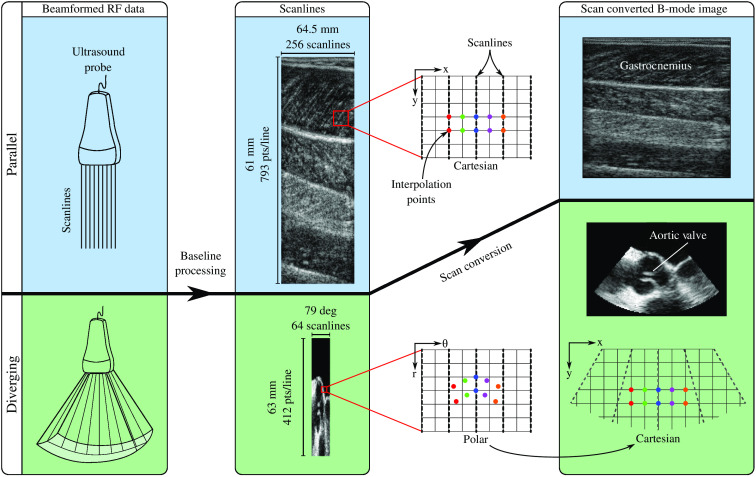
Illustration of a simplified ultrasound image acquisition pipeline highlighting the differences in the scan conversion step for linear and diverging ultrasound scanlines. *In vivo* scanline data are shown to demonstrate the spatial effects of scan conversion. (Top) The posterior side of a human lower leg was scanned using an ultrasound probe with 256 parallel scanlines and 793 points along each scanline. The gastrocnemious and soleus muscles are clearly visible in the image. The scanlines and the interpolation points (colored) for generating the B-mode image lie on a grid. (Bottom) An ultrasound image of a human aortic valve was acquired using a 3D TEE probe with 64×48 diverging scanlines, with 412 points along each scanline. Here, we show a single 2D slice out of the 48. The scanlines lie on a grid in polar coordinates, however the interpolation points (colored) for generating the B-mode image lie on a grid in Cartesian coordinates. Mapping these interpolation points to polar coordinates results in a non-uniform spacing between interpolation points. Scan conversion is often performed in polar coordinates, then the interpolated points are mapped back into Cartesian coordinates.

In contrast, with diverging scanlines, the scanline data form a grid in polar coordinates, but the clinician is often interested in the Cartesian space representation for ease of spatial reasoning. Figure 1 shows the arrangement of scanlines in polar and Cartesian coordinates. The desired interpolation points (colored) lie on the image grid in Cartesian space. It is noteworthy that with diverging scanlines, the density of observations in Cartesian space gets sparser as the distance from the ultrasound transducer (r) increases. In practice, when using linear interpolation methods, scan conversion for diverging scanlines is performed in polar coordinates, where the observation points lie on a grid. The interpolated intensity values are then simply displayed in Cartesian coordinates. However, this method ignores the increasing distance between observations due to the mapping between polar and Cartesian spaces, which can be problematic especially when the beam spacing is sparse, or if the clinician wants to zoom in on regions that are further away from the probe. To achieve better accuracy, the interpolation method should account for the change in inter-beam distance, capturing the underlying process better. In Sec. 2.3, we discuss how this can be achieved by selecting a different kernel length per dimension in the GP covariance function.

### 2.3 Covariance Functions

The covariance function (i.e., kernel) captures the spatial dependence between observations. There are various kernels that can be used to compute the covariance matrix, and a comprehensive list can be found in Ref. 17. In this work, we consider two covariance functions: the squared exponential (SE) and the Matérn.

Kernels usually have a parameter that controls the characteristic length scale of the dependence between observations. It is possible to set a different length scale for each dimension of the data, which is useful when dealing with a non-isometric sampling density, such as in the case of ultrasound imaging with diverging scanlines.

#### 2.3.1 Squared exponential kernel

The SE covariance function, also known as the radial basis function kernel, is an infinitely differentiable function, which results in a smooth process. The SE covariance function with separate length scales for each dimension is expressed as

$$k(x_{i},x_{j}|\theta )=\sigma^{2}\;\exp[-\overset{2}{\underset{n=1}{\sum }}\frac{{\left(x_{i,n}-x_{j,n}\right)}^{2}}{2l_{n}^{2}}],$$ (4)

where θ is the function parameters (σ and $l_{n}$), $\sigma^{2}$ is the signal variance, $l_{n}$ is the characteristic length scale for dimension n, and $x_{i,n}$ is the n’th component of the i’th observation.

In this work, we will denote the length scale along the scanlines as $l_{r}$, and normal to the scanlines as $l_{\theta }$.

#### 2.3.2 Matérn kernel

The Matérn autocovariance function has the form

$$k(x_{i},x_{j}|\theta )=\sigma^{2}\frac{2^{1-\nu }}{\Gamma (\nu )}{\left(\frac{\sqrt{2\nu }}{l}r\right)}^{\nu }K_{\nu }(\frac{\sqrt{2\nu }}{l}r),$$ (5)

where Γ is the gamma function, $K_{\nu }$ is the modified Bessel function of the second kind, r is the distance between $x_{i}$ and $x_{j}$, l is the characteristic length scale, and ν is the smoothness parameter. In D dimensions, the distance metric is expressed as

$$r(x_{i},x_{j})=\sqrt{\overset{D}{\underset{n=0}{\sum }}\frac{{\left(x_{i,n}-x_{j,n}\right)}^{2}}{l_{n}^{2}}}.$$ (6)

According to Ref. 19, the strong smoothness assumptions of the SE covariance function are unrealistic for modeling many physical processes, and the Matérn class of covariance functions is recommended instead.^17^ The Matérn covariance function is ⌈v⌉−1 times differentiable. In general, ν is chosen to be a half-integer, which reduces Eq. (5) to an exponential multiplied by a polynomial, and the spectral density becomes rational.^19^ When ν=1/2, the Matérn covariance function reduces to the exponential covariance function, and as ν→∞ the kernel converges to the SE covariance function. In the literature, ν is usually set to 3/2 or 5/2.^17^

We tested both the SE kernel and the Matérn covariance function with ν=3/2 and 5/2 in our experiments. The Matérn covariance function with ν=3/2 yielded the most accurate interpolation, therefore all results reported in Sec. 3 are computed using this kernel.

### 2.4 Ground Truth Data and Measuring Interpolation Accuracy

Quantifying improvement in image quality is a challenging task in the absence of ground truth data. In this study, the original ultrasound scanline data are treated as the ground truth. The interpolation methods outlined are then only applied to a subset of the scanlines, and the resulting estimates are then compared with the excluded scanlines to measure the interpolation accuracy. We will refer to this as leave-N-out in Sec. 3. We compare the performance of commonly used interpolation methods (nearest neighbor, bilinear, and cubic spline interpolation) against the performance of GP regression, where the mean absolute error (MAE) and the peak signal-to-noise ratio (PSNR) between the ground truth and the interpolated images are measured.

The MAE is defined as

MAE=1I∑iI|Zi*−Z^i|, (7)

where I is the total number of pixels in the ultrasound image, $Z_{i}^{*}$ is the pixel intensities in the ground truth image, and ${\hat{Z}}_{i}$ is the pixel intensities in the interpolated image.

Assuming that pixel intensities are normalized to R:[0,1], the PSNR is defined as

$$\mathrm{PSNR}=10\;\log_{10}(\frac{1}{\mathrm{MSE}}),$$ (8)

where

MSE=1I∑iI(Zi*−Z^i)2. (9)

The best interpolation method should ideally have the highest PSNR and lowest MAE and MSE among the interpolation methods tested.

### 2.5 Optimizing the Kernel Length Scale

In general, the kernel parameters for a GP are determined by minimizing the log marginal likelihood,

$$\log\;p(y|X)=-\frac{1}{2}y^{T}{\left(K+\sigma_{n}^{2}I\right)}^{-1}y\;-\frac{1}{2}\;\log|K+\sigma_{n}^{2}I|-\frac{N}{2}\;\log\;2\pi ,$$ (10)

where N is the number of observations. This is equivalent to minimizing the mean squared error (MSE). We optimize the kernel parameters through a constrained optimization problem with the MSE metric as the cost function, described as

minln 1I∑iI(Zi*−Z^iGP)2s.t.  ln>0,  n=α,r, (11)

where $Z_{i}^{*}$ is the ground truth pixel intensity and ${\hat{Z}}_{i}^{\mathrm{GP}}$ is the GP estimate. In our optimization studies, we noticed that the landscape is convex, however, the gradients near the optimum are small.

**Fig. 2:**
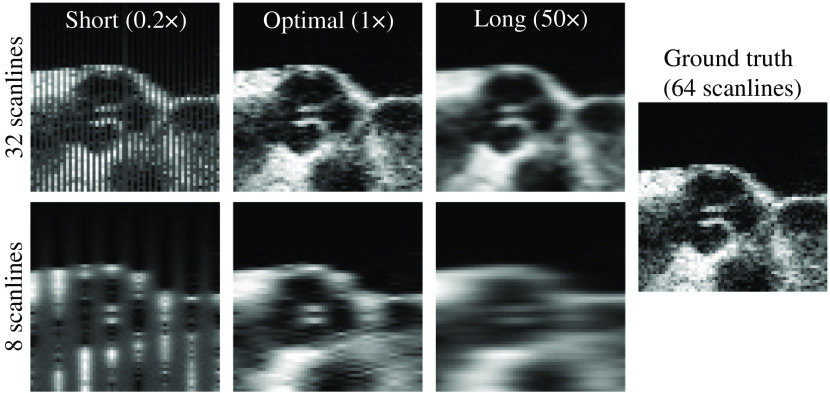
Incorrect choice of the kernel length can lead to reconstruction errors, such as missing data (shorter than optimal) or blurring (longer than optimal). Ground truth shown on the right uses all 64 scanlines, whereas the reconstructions on the left use 32 (top row) and 8 (bottom row) scanlines. When the kernel length is too short, any regression point that lies too far from an observation quickly approaches the mean of the GP.

Figure 2 shows how choosing a larger than optimal length scale results in blurring, while shorter length scales lead to striated mean-valued regions between the scanlines. Using a sub-optimal length scale results in a decrease in the PSNR.

### 2.6 Reducing Computational Cost—Patched GP Regression

GP regression has $O(N^{3})$ memory and $O(N^{2})$ time complexity, where N is the number of observations,^17^ which is perhaps the main reason why it has not been widely adopted in clinical imaging. Recent work^20^ has pushed exact GP training to over one million data points using multi-GPU parallelization and methods such as linear conjugate gradients. However, medical images can contain tens of millions of pixels or voxels and medical applications generally require real-time performance.

The main bottleneck in GP regression is in inverting the covariance matrix in Eq. (2). The covariance function gives a measure of the spatial dependence between observations. It is noteworthy that with certain covariance functions (such as the ones used in this work), the covariance function quickly decays as the distance between observation pairs increases. Therefore, outside of a local region, the contributions from other observations can practically be ignored without a loss in regression accuracy. We exploit this behavior to reduce the computational demand of GP regression. The scanline data are subdivided into overlapping patches and GP regression is evaluated separately for each patch.

**Fig. 3:**
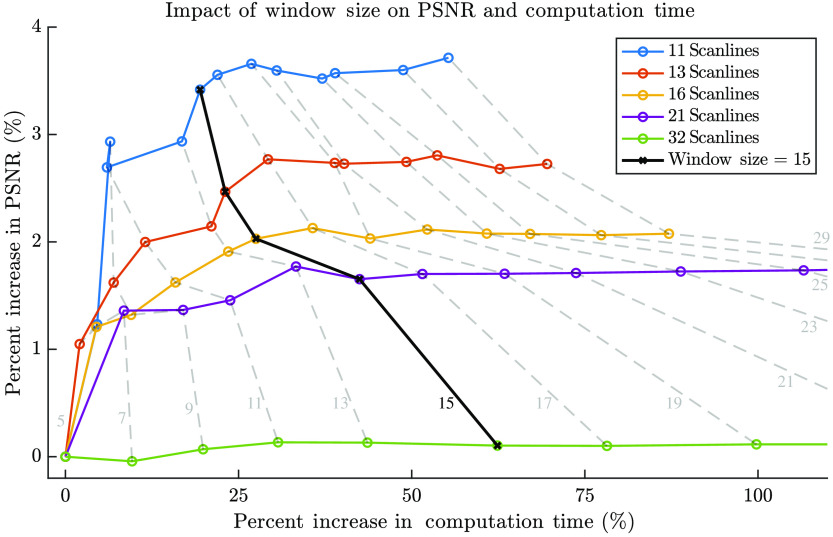
Results of the window size study on the phased-array scanline data, showing the percent increase in computation time and PSNR as the window size is increased from 5 to 29. Bold black line shows the 15 observations we selected. The colored lines represent different levels of decimation of the scanlines. The computation times reported here are the mean of 10 measurements per window size.

Experimenting with a range of window sizes, we determined that a window size of 15 observations is optimal. Figure 3 shows the percent increase in computation time and PSNR as the window size is increased from 5 to 29. Bold black line indicates the 15 observation window size we selected. The colored lines represent different levels of decimation of the scanlines. This plot is for the phased-array dataset. We can see that increasing the window size beyond 15 yields negligible or no increase in PSNR at the cost of increased computation time. It is noteworthy that the windowed GP regression can be parallelized on a GPU or field programmable gate arrays (FPGA) to process the entire image in a single pass.

Furthermore, the computation time and costs can be significantly reduced by pre-computing the matrix $K_{*}^{T}{\left(K+\sigma_{n}^{2}I\right)}^{-1}$ in Eq. (2) and $K_{**}$ in Eq. (3). For a given probe and imaging depth, the observation and interpolation locations are not going to change, therefore, these matrices can be pre-computed to avoid performing the costly Cholesky decomposition with each regression. This reduces the computational complexity to just O(NM) for the matrix-vector multiplication in Eq. (2), where M<N is the number of interpolation locations.

## 3 Experiments and Results

*In vivo* scanline data from two different ultrasound imaging systems were acquired. A linear-array ultrasound probe with parallel scanlines (LV8-4L65S-3, TELEMED Ltd., Vilnius, Lithuania) was used to acquire 2D images of the lower leg of a healthy male subject at a 61-MHz imaging depth and 6-MHz frequency. The 256 scanlines with 793 observations along each scanline are shown in Fig. 1(top). In another experiment, 3D scanline data of a human heart was acquired at 63-mm imaging depth and 7-MHz frequency using a phased-array 3D transesophageal echocardiography (TEE) probe (X7-2t transducer connected to an iE33 ultrasound imaging system, Philips Healthcare, Andover, Massachusetts, United States). A 2D slice (out of 48) from the 3D dataset is shown in Fig. 1(bottom), containing 64 scanlines and 412 observations along each scanline. Both studies were approved by the Harvard University Institutional Review Board (IRB) and the Boston Children’s Hospital IRB.

The observation noise $\sigma_{n}^{2}$ for GP regression was set to $2\times 10^{-3}$ for the linear probe, and $8\times 10^{-3}$ for the phased-array probe through hyperparameter optimization. Computations used the image processing, parallel computing, and the statistics and machine learning toolboxes in MATLAB (The MathWorks, Inc., Natick, Massachusetts, United States).

### 3.1 Parallel Scanlines

**Fig. 4:**
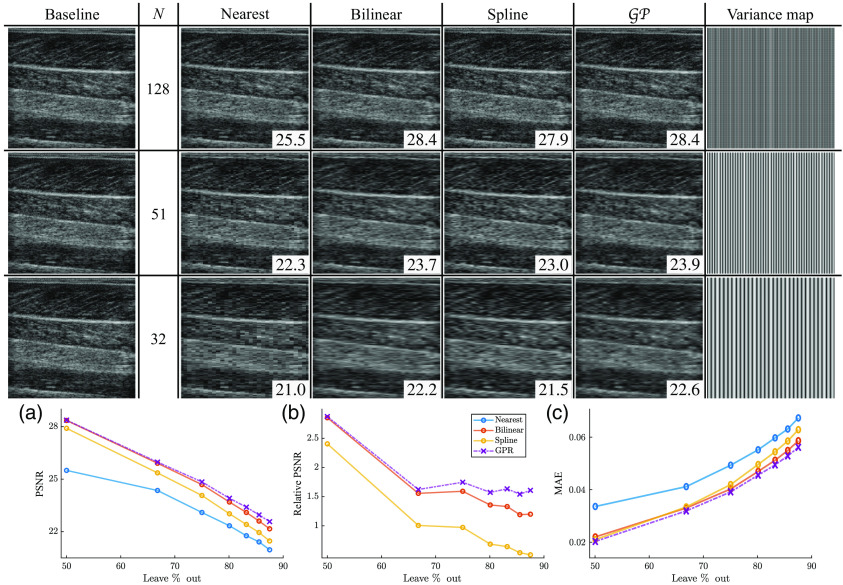
(Top) Scan converted B-mode images of the parallel scanline dataset. Leftmost column shows the ground truth data with 256 scanlines. N indicates the number of scanlines used for interpolation, and the next four columns show the resulting B-mode images using four interpolation methods (nearest, bilinear, spline, and GP). Rightmost column shows the normalized variance matrix generated during GP regression, where darker regions indicate higher confidence in the estimates. PSNR scores are overlaid on each image. (Bottom) Interpolator performance for scan conversion of parallel scanlines: (a) absolute PSNR scores (higher is better), (b) relative PSNR scores (computed by subtracting the lowest scores from the rest, higher is better), and (c) MAE scores (lower is better).

Figure 4 shows the interpolated B-mode images for three different leave-N-out studies. Leftmost column shows the ground truth data. The variance map on the right shows the normalized values of the variance reported by GP regression from Eq. (3), where darker regions indicate smaller variance (i.e., higher confidence). The normalization coefficient was determined across the entire image set.

It is worthy to note that the amount of uncertainty increases as the number of scanlines is reduced. Figure 4(a) shows the absolute PSNR scores, Fig. 4(b) shows the relative PSNR scores (computed by subtracting the lowest scores from the rest), and Fig. 4(c) shows the MAE scores. Nearest neighbor interpolation has the largest error as expected, and GP regression has the lowest error and highest PSNR.

As the number of observations (scanlines) is reduced, we see a wider separation in performance between GP regression and the other interpolation methods. Looking at the relative PSNR scores, we see that the scores for the bilinear and spline methods have a negative slope that tend toward the performance of the nearest neighbor interpolation. However, GP regression maintains a constant separation in metrics from the baseline method (nearest neighbor).

### 3.2 Diverging Scanlines

**Fig. 5:**
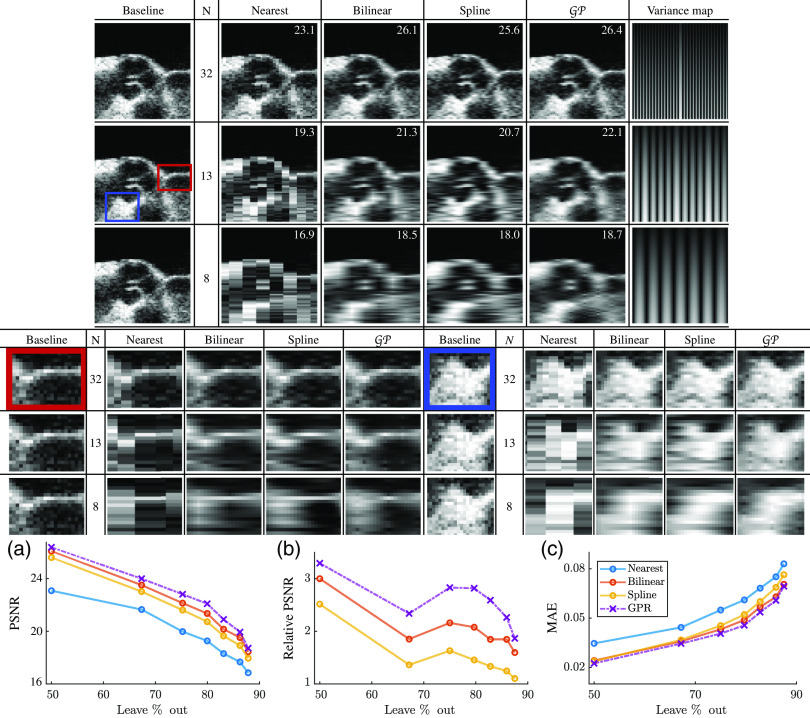
(Top) Scan converted B-mode images of the diverging scanline dataset, presented in polar coordinates. In the top three rows, leftmost column shows the ground truth data with 64 scanlines. N indicates the number of scanlines used for interpolation, and the next four columns show the resulting B-mode images using four interpolation methods (nearest, bilinear, spline, and GP). PSNR scores are overlaid on each image. Rightmost column shows the normalized variance matrix generated during GP regression. Bottom three rows show a detailed view of the red and blue regions of interest. (Bottom) Interpolator performance for scan conversion of diverging scanlines: (a) absolute PSNR scores (higher is better), (b) relative PSNR scores (computed by subtracting the lowest scores from the rest, higher is better), and (c) MAE scores (lower is better).

Figure 5 shows the interpolated B-mode images for three different leave-N-out studies in polar coordinates. Leftmost column shows the ground truth data. The variance map on the right shows the normalized values of the variance reported by GP regression from Eq. (3). The variance maps shown in Fig. 5 indicate that the amount of uncertainty increases as the number of scanlines is reduced. The variance also increases along the scanlines due to the 1/r scaling of the $l_{\alpha }$ parameter. Figure 5(a) shows the absolute PSNR scores, Fig. 5(b) shows the relative PSNR scores (computed by subtracting the lowest scores from the rest), and Fig. 5(c) shows the MAE scores. Nearest neighbor interpolation has the largest error as expected, and GP regression has the lowest error and highest PSNR. GP regression exhibits a larger increase in performance for diverging scanlines compared to the linear scanlines.

We observe an even wider separation (compared to parallel scanline results) in performance between GP regression and the other interpolation methods as the number of observations (scanlines) is reduced. Looking at the relative PSNR scores, we see that the scores for the bilinear and spline methods again have a negative slope that tend toward the performance of the nearest neighbor interpolation. Differently from the parallel case, the relative PSNR score for GP regression has a peak around 13 scanlines, then rapidly decreases near the performance of bilinear interpolation. However, we should notice that this occurs when the number of scanlines is <15, which is the window size we determined previously when analyzing the GP kernel range of influence. This suggests that we simply do not have enough data points to accurately infer the underlying distribution of the data, thus GP regression performance drops rapidly.

**Fig. 6:**
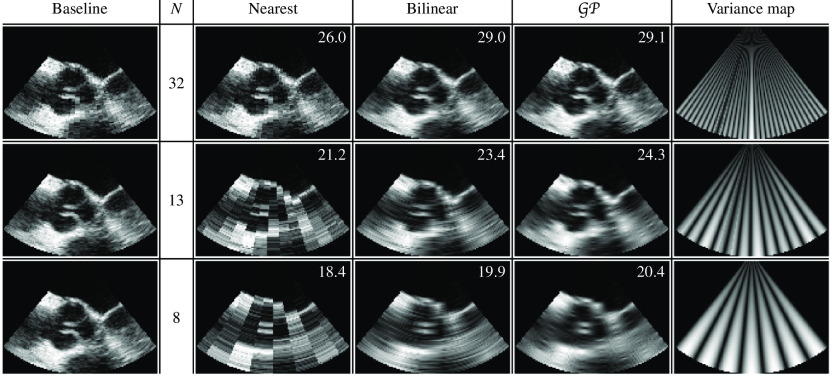
Scan converted B-mode images of the diverging scanline dataset, presented in Cartesian coordinates. Leftmost column shows the baseline image with 64 scanlines. PSNR scores are overlaid on each image. Rightmost column shows the normalized variance map generated during GP regression.

Figure 6 shows the interpolated B-mode images for three different leave-N-out studies in Cartesian coordinates. Leftmost column shows the baseline data, which was generated by converting the scanlines from polar coordinates to Cartesian coordinates using bilinear interpolation, and all 64 of the available scanlines were used in the scan conversion. We also tested cubic interpolation and GP regression to generate the baseline, which resulted in similar PSNR and MAE metrics. The variance map on the right shows the normalized values of the variance reported by GP regression from Eq. (3). We can see that the amount of uncertainty increases as the number of scanlines is reduced. The variance also increases along the scanlines due to the distance-dependent (1/r) scaling of the $l_{\alpha }$ parameter. At higher decimation rates with N=13 and N=8, the B-mode images generated by GP regression are visibly clearer compared to the other interpolation methods. Bilinear interpolation results exhibit the laterally elongated features that is characteristic to ultrasound images seen in commercial ultrasound systems.

### 3.3 Relationship between Scanline Properties and Optimal Kernel Lengths

Optimal kernel lengths were calculated by minimizing the problem defined in Eq. (11). This function had to be minimized for each different number of scanlines used, which is a computationally expensive process. However, in this particular application, since observations lie on a fixed grid, it should be possible to determine a relationship between the grid parameters and the optimal kernel lengths. This relationship can be then used to directly calculate the kernel length for a given grid, without having to run an expensive optimization cycle.

In the case of parallel scanlines, the distance between observations along the scanlines, Δr, and the distance between the scanlines, |rΔα|, are constants that are determined by the ultrasound probe and imaging settings.

In the case of diverging scanlines, the regression variance along the rays r should be constant, since the distance between observations is constant. However, as the scanlines diverge, since there are less observations per unit area, the variance across scanlines (along the α axis) should increase. This can be achieved by scaling $l_{\alpha }$ by 1/r, which reduces the characteristic length scale in the radial axis, thus, increasing the uncertainty of the pixels in between the scanlines.

Inspecting of the optimized kernel lengths, we noticed the following relationships between the kernel lengths and the physical parameters of the ultrasound scanlines:

1. For parallel scanlines,
  $$l_{\alpha }=\frac{1}{\sqrt{3}}\Delta \alpha ,$$ (12)
  $$l_{r}=k_{N}\Delta r,$$ (13)
  where Δα and Δr are the (standardized) distances between observations (i.e., voxel size), and $k_{N}$ is the reduction factor for leave-N-out studies. For example, if only N/4 scanlines are used in the interpolation, then $k_{N}=4$.
2. For diverging scanlines,
  $$l_{\alpha |{\overset{¯}{r}}_{i}}=\frac{1}{\sqrt{3}}\frac{\left|\overset{¯}{r}\right|}{2{\overset{¯}{r}}_{i}}\Delta \alpha ,$$ (14)
  $$l_{r}=k_{N}\Delta r,$$ (15)
  where $\left|\overset{¯}{r}\right|$ is the overall (standardized) scanline length, and ${\overset{¯}{r}}_{i}$ is the depth of the i’th observation along the scanline.

It is worthy to note that we have not yet tested if these kernel length scale relationships hold true across different anatomy, scan depths, and ultrasound probes.

## 4 Discussion

In this paper, we investigated the use of GP regression for ultrasound scanline interpolation. We analyzed *in vivo* ultrasound data acquired using both linear and phased-array probes to validate the performance of GP regression. Using leave-N-out studies, we qualitatively and quantitatively showed that GP regression leads to better B-mode conversion than other interpolation methods, indicated by the higher PSNR and lower MAE scores. We greatly reduced the computation time through patched computation of the inverse of the covariance function. In our implementation, the average time to estimate the value of one data point was 0.3 ms. It should be possible to enable real-time execution of GP regression through an optimized implementation. The covariance matrix can also be precomputed, significantly reducing the computational complexity of the problem. Computational time can be further reduced by relaxing the requirements on exact regression, and taking advantage of the Kronecker structure of gridded data.^21^

We demonstrated that GP regression leads to better scanline conversion using clinical data. We collected ultrasound scanline data from two distinct parts of the anatomy using a linear and phased-array ultrasound probe. The leg dataset exhibits high-frequency texture and has a dense set of scanlines, whereas the cardiac dataset comprises uniform patches of dark (blood) and bright (muscle) regions and has a sparse set of scanlines. The PSNR and MAE scores for GP regression are better than those for nearest, bilinear, and spline interpolation across both datasets.

Using GP regression, it may be possible to use fewer scanlines while maintaining the same image quality. This can reduce system complexity or help increase the frame rate by reducing number of transmitted beams.

Beyond improving scan conversion accuracy, our method can be further beneficial in downstream tasks, such as image registration. The uncertainty estimate of GP regression can be used to improve ultrasound image registration accuracy. This technique was successfully demonstrated for MRI registration.^16^

One open question in our study is whether the kernel length relationships that we identified hold for different anatomy and ultrasound probes. We plan to address this in our future work.

Choosing a covariance function prescribes a prior on the spatial variation of the data. Even though the Matérn 3/2 function resulted in lower errors compared to the SE kernel, use of other covariance functions should be investigated as well.

In this work, we applied GP regression to scanline data that was acquired after log compression to an 8-bit range. However, this compresses and distorts the dynamic range of the ultrasound transducer. We expect performing regression prior to these post-processing steps will lead to better scanline conversion. In combination with tone mapping techniques and high-dynamic range ultrasound imaging methods,^22^ the ultrasound images presented to the clinician can be further improved.
